# An Esterase-Responsive SLC7A11 shRNA Delivery System Induced Ferroptosis and Suppressed Hepatocellular Carcinoma Progression

**DOI:** 10.3390/pharmaceutics16020249

**Published:** 2024-02-08

**Authors:** Hui Zhang, Jianguo Wang, Xiaonan Xiang, Chang Xie, Xinfeng Lu, Haijun Guo, Yiyang Sun, Zhixiong Shi, Hongliang Song, Nasha Qiu, Xiao Xu

**Affiliations:** 1 The Fourth School of Clinical Medicine, Zhejiang Chinese Medical University, Hangzhou 310053, China; zh0319@zcmu.edu.cn (H.Z.); wangjianguo8059@zju.edu.cn (J.W.); luxinfeng@zcmu.edu.cn (X.L.); sunyiyang@zcmu.edu.cn (Y.S.); shl@zcmu.edu.cn (H.S.); 2 Key Laboratory of Integrated Oncology and Intelligent Medicine of Zhejiang Province, Affiliated Hangzhou First People’s Hospital, Hangzhou 310006, China; xnxiang@zju.edu.cn (X.X.); xiechang@stu.hznu.edu.cn (C.X.); guohaijun431@hotmail.com (H.G.); shizx@zju.edu.cn (Z.S.); 3 School of Clinical Medicine, Zhejiang Chinese Medical University, Hangzhou 310053, China; 4 Zhejiang University School of Medicine, Hangzhou 310058, China; 5 School of Clinical Medicine, Hangzhou Normal University, Hangzhou 311121, China

**Keywords:** ferroptosis, hepatocellular carcinoma gene therapy, esterase-responsive polymer, SLC7A11 shRNA

## Abstract

Ferroptosis has garnered attention as a potential approach to fight against cancer, which is characterized by the iron-driven buildup of lipid peroxidation. However, the robust defense mechanisms against intracellular ferroptosis pose significant challenges to its effective induction. In this paper, an effective gene delivery vehicle was developed to transport solute carrier family 7 member 11 (SLC7A11) shRNA (shSLC7A11), which downregulates the expression of the channel protein SLC7A11 and glutathione peroxidase 4 (GPX4), evoking a surge in reactive oxygen species production, iron accumulation, and lipid peroxidation in hepatocellular carcinoma (HCC) cells, and subsequently leading to ferroptosis. This delivery system is composed of an HCC-targeting lipid layer and esterase-responsive cationic polymer, a poly{N-[2-(acryloyloxy)ethyl]-N-[p-acetyloxyphenyl]-N} (PQDEA) condensed shSLC7A11 core (G−LPQDEA/shSLC7A11). After intravenous (i.v.) injection, G−LPQDEA/shSLC7A11 quickly accumulated in the tumor, retarding its growth by 77% and improving survival by two times. This study is the first to construct a gene delivery system, G−LPQDEA/shSLC7A11, that effectively inhibits HCC progression by downregulating SLC7A11 expression. This underscores its therapeutic potential as a safe and valuable candidate for clinical treatment.

## 1. Introduction

Hepatocellular carcinoma (HCC) is the most common cause of lethal mortality of adult males under 60 years old in China. Alarmingly, its incidence is increasing globally [1].

In recent years, remarkable advancements have been achieved in the therapeutic landscape of HCC, with notable improvements in surgical resection techniques, tumor ablation strategies, loco-regional therapies, targeted interventions, immunotherapy, et al. [2,3,4,5]. Due to the high metastasis and recurrence rates, conventional therapies are unsatisfactory [6,7,8]. This is because most chemotherapeutic agents are designed to induce HCC apoptosis [9,10], while tumor cells develop anti-apoptotic mechanisms and are resistant to common therapeutics [11]. Hence, it is of great importance to seek novel strategies against HCC.

Ferroptosis, characterized by intracellular iron-driven catalysis, lipid peroxidation, and redox balance disruption [12,13,14,15], has emerged as a novel avenue for HCC intervention. This is especially pertinent for aggressive tumors that are resistant to apoptosis induction through conventional therapeutic modalities [12,13].

Conceptually, tumor cells exhibit heightened metabolic activity compared to normal cells, meeting the requirements for their rapid proliferation. This heightened metabolic state exposes them to increased levels of reactive oxygen species (ROS), leading to lipid peroxidation and ferroptosis [16]. Additionally, cancer cells often demand a high level of iron, sensitizing them to ferroptosis [10]. GPX4 (glutathione peroxidase 4) is a lipid hydroperoxidase that is critical to tumor survival. It is reported that a loss of GPX4 function results in tumor cell ferroptosis and prevents tumor relapse [11].

Conventional strategies for ferroptosis induction involve small compounds like erastin, sorafenib [17,18], sulfasalazine [19], artemisinin, et al. [20,21]. These small molecules are easily degraded, and their half-lives are usually short, limiting tumor accumulation [22]. Furthermore, the poor specificities and repeated administrations of these small molecules lead to adverse effects on normal tissues, thus hindering the long-acting ferroptosis induction.

Recent studies have demonstrated that system Xc−, a transporter for amino acids, orchestrates the influx of cystine while exporting glutamate. This process leads to the production of antioxidant glutathione (GSH), which interacts with GPX4 to decrease phospholipid hydroperoxides (PL-OOH) and produce the corresponding alcohols (P-LOH) [12]. Ultimately, this helps to protect the integrity of cell membranes. The subunit of system Xc−, SLC7A11, has been shown to play a crucial role in regulating the activity of system Xc−. Thus, downregulating SLC7A11 was reported to suppress glutathione synthesis and trigger ferroptosis [19,23]. Most studies of SLC7A11 gene therapy usually incorporated chemo drugs [24,25,26,27], while solely gene therapy by targeting SLC7A11 has yet to be achieved, probably due to unspecific and inefficient gene silencing.

In our previous work, we developed esterase-responsive charge-reversal polymers with excellent transfection efficiencies in tumor cells [28,29] and tumor-associated macrophages [30]. P-acetyloxybenzyl groups in the cationic polymer (PQDEA) are easily hydrolyzed by esterase to generate poly (acrylic acid) through self-catalysis, resulting in a cationic to anionic charge reversal, leading to shSLC7A11 release from polyplexes. These polymers are readily hydrolyzed by intracellular esterases, yielding a positive-to-negative charge conversion for expeditious shSLC7A11 release, facilitating gene expression.

**Scheme 1:**
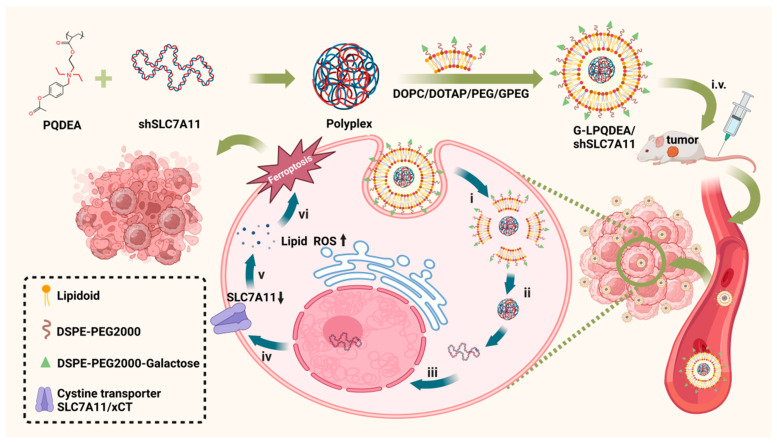
Fabrication of G−LPQDEA/shSLC7A11 nanoparticles and their therapeutic mechanisms on LM3 subcutaneous tumors. (**i**,**ii**) PQDEA efficiently condensed shSLC7A11 into polyplexes, which are readily coated with DC-Chol/DOPC lipids through hydrophobic interactions, resulting in the formation of lipidic esterase-responsive polyplexes (LPQDEA/shSLC7A11). They were then inserted with DSPE-PEG2000 and DSPE-PEG2000-galactose-decorated lipid layer to obtain the finalized G−LPQDEA/shSLC7A11. When injected intravenously into nude mice bearing LM3 tumors, G−LPQDEA/shSLC7A11 accumulated in the tumors and was internalized by tumor cells rich in esterase. The lipid layer was then peeled off to expose the PQDEA/shSLC7A11 core, undergoing cellular esterase hydrolyzation to realize positive-to-negative charge conversion and shSLC7A11 release. (**iii**–**vi**) The released shSLC7A11 entered the nucleus to degrade SLC7A11 mRNA and further suppress SLC7A11 expression, inhibiting GSH synthesis and GPX4 expression. This ultimately led to ferroptosis.

In this study, the SLC7A11 shRNA (shSLC7A11) gene delivery system G−LPQDEA/shSLC7A11 was developed using esterase-responsive polymers, PQDEA, and HCC-targeting long-circulating liposomes (galactose-decorated DSPE-PEG, cationic lipid DOTAP, and phospholipid DOPC). G−LPQDEA/shSLC7A11 nanoparticles were quickly internalized by HCC cells and easily escaped from lysosomes. The intracellular esterase hydrolyzes PQDEA to generate an anionic polymer—polyacrylic acid—and releases shSLC7A11, which enters the nucleus to degrade SLC7A11 mRNA, further inhibiting SLC7A11 expression. The downregulation of SLC7A11 leads to the restriction of GSH biosynthesis, GPX4 inactivation, and lipid peroxide accumulation, which finally induces cell ferroptosis. G−LPQDEA/shSLC7A11 treatment sufficiently suppressed HCC tumor growth and extended the survival of the studied mice (Scheme 1).

## 2. Materials and Methods

### 2.1. Materials

The PCR primers were designed and synthesized by Qingke (Hangzhou, China). The SYBR green PCR Master Mix was acquired from Thermo Scientific (Waltham, MA, USA). GPX4 and SLC7A11 antibodies were acquired from Proteintech Co., Ltd. (Wuhan, China). A Label IT^®^ Nucleic Acid Labeling Kit, Cy^®^5 was acquired from Shanghai Universal Biotech Co., Ltd. (Shanghai, China). A Lipid Peroxidation MDA Assay Kit, CCK-8 Cell Proliferation and Cytotoxicity Assay Kit, and GSH detection kit were purchased from Beyotime (Shanghai, China). A Phen GreenSK, Diacetate, and ROS detection kit was purchased from Invitrogen™ (Beijing, China). LysoTracker Green and Hoechst 33342 were acquired from Invitrogen (Carlsbad, CA, USA). Cell culture media, Dulbecco’s modified Eagle’s medium (DMEM), fetal bovine serum, and Trypsin were purchased from Gibco (Thermo Fisher Scientific, Shanghai, China). 4-(2-hydroxyethyl)-1-piperazine ethane sulfonic acid (HEPES) was obtained from Biowest (Nuaillé, France).

### 2.2. Methods

#### 2.2.1. Patients and Specimens

Between 2015 and 2018, 226 normal liver tissues and 230 HCC tissues were collected with informed consent from patients at First Affiliated Hospital, College of Medicine, Zhejiang University. The overall survival rate (OS) was measured from the time of surgery to death or the last follow-up. The study of patient specimens was approved by Zhejiang University’s Ethics Committee.

#### 2.2.2. Immunohistochemical (IHC) Analysis

In a tissue microarray (TMA), 226 normal liver and 230 HCC tissues were assembled and analyzed with an anti-SLC7A11 antibody (1:1000, Proteintech, Wuhan, China).

#### 2.2.3. Cell Lines and Cell Culture

LM3 cells, Hepa 1–6 cells, and AML12 cells were obtained from the American Type Culture Collection and cultured in Dulbecco’s modified Eagle’s medium (DMEM) containing 10% heat-inactivated fetal bovine serum, and 1% penicillin–streptomycin. The cells were cultured at 37 °C in a humidified atmosphere of 5% CO_2_.

#### 2.2.4. Preparation and Characterization of Polyplexes with Different N/P Ratios

shSLC7A11 was diluted to a concentration of 40 µg mL^−1^ in the HEPES buffer (10 mM, pH = 7.3). PQDEA was dissolved at 50 mg/mL in DMSO. Then, 50 μL of the stock solution was dissolved in the HEPES buffer solution to attain the required concentrations based on the specified N/P ratios (N/P = 1, 3, 5, 7, 9, 11, 13, 15, 17, 19). Then, the PQDEA solution was mixed with an equivalent volume of the shSLC7A11 solution for 10 s, and then incubated for 30 min at room temperature. The size distributions and zeta potentials of PQDEA/shSLC7A11 nanoparticles in the HEPES buffer with various N/P ratios were determined with Zetasizer Nano-ZS (Malvern Instruments, Malvern, UK). TEM (JEM-1200EX) was used to visualize polyplex morphology after uranyl acetate staining.

#### 2.2.5. Fabrication and Characterization of the PEF-GPEG-Decorated Lipopolyplexes (G−LPQDEA/shSLC7A11)

A PEG-GPEG-decorated polyplex was prepared according to the thin-film hydration method. The fabrication protocol is outlined as follows: dissolve DOPC (2.36 mg, 0.03 mM), DOTAP (0.70 mg, 0.01 mM), PEG (1 mg, 0.005 mM), and GPEG (1 mg, 0.005 mM) in 1 mL of chloroform with continuous stirring, followed by evaporation to yield a thin lipid film. The lipid solution was stirred overnight at room temperature and sonicated for 10 min prior to use. The lipopolyplex solution was then filtered through a 0.22 µm nylon filter to remove non-encapsulated drug aggregates.

Subsequently, the PQDEA/shSLC7A11 polyplex solution at an N/P ratio of 7 was added to the lipid solution dropwise to obtain G−LPQDEA/shSLC7A11. The morphology of G−LPQDEA/shSLC7A11 was visualized with TEM after staining with uranyl acetate.

#### 2.2.6. Cell Viability Assay

The CCK-8 Assay Kit was used to evaluate the cytotoxicity of the polyplexes (Varioskan Flash, Thermo Scientific, Shanghai, China).

#### 2.2.7. Measurement of Cellular Labile Iron

The intracellular labile iron levels were assessed using a Phen Green™ SK diacetate (PGSK) metal sensor. Typically, LM3 cells and Hepa 1–6 cells were placed in 20 mm confocal dishes with glass bottoms at a density of 2 × 10^5^ per well. After being washed with PBS, they were treated with 5 μM PGSK in PBS for 20 min at 37 °C in the dark. After the incubation period, the cells were rinsed with PBS. Subsequently, the cells were imaged using a confocal laser scanning microscope (Nikon A1) equipped with a 60× oil lens.

#### 2.2.8. ROS Analysis

The LM3 cells and Hepa 1–6 cells were seeded in 20 mm glass-bottomed confocal dishes at 2 × 10^5^ cells per well for 24 h. The dishes were loaded with various intervention reagents; after a 48 h incubation, the cells were washed with PBS and were then stained with a 10 μM C11-BODIPY (581/591) probe in the complete medium for 30 min. Subsequently, nuclei were stained with Hoechst 33342. An Olympus IX81 Inverted Microscope was utilized for imaging. The O-BODIPY was observed using excitation/emission wavelengths of 488Ex/510Em, whereas the R-BODIPY was visualized at excitation/emission wavelengths of 581 Ex/591 Em.

#### 2.2.9. GSH Analysis

GSH levels were assessed utilizing a GSH detection kit according to the manufacturer’s guidelines. Briefly, 60 × 10^5^ LM3 and Hepa 1–6 cells were seeded into 6-well cell culture plates and cultured for 24 h. Subsequently, cells were then treated with G−LPQDEA/shSLC7A11 for 48 h, collected and resuspended in reagent I, and subjected to three cycles of freezing and thawing with liquid nitrogen. After centrifugation at 8000 rpm, reagent II and reagent III were mixed with the supernatant, and GSH levels were determined using a multifunctional enzyme marker at a wavelength of 412 nm.

#### 2.2.10. Lipid Peroxidation (MDA) Assay

Cells (Hepa 1–6, LM3 cells) were incubated with G−LPQDEA/shSLC7A11 for 24 h and then collected and centrifuged at 1000 rpm for 5 min. Afterwards, the cells were subjected to sonication and then centrifuged for 10 min at 12,000 rpm. Using a Lipid Peroxidation (MDA) Assay Kit, the resultant supernatant was mixed with thiobarbituric acid and incubated at 95 °C for 60 min. The samples were then chilled on ice for a duration of 10 min, and analyzed with a microplate reader.

#### 2.2.11. In Vivo Experiments

The experimental protocols of the animal studies were approved by Zhejiang University’s Institutional Animal Care and Use Committee. Female BALB/c nude mice (7–8 weeks old) were purchased from the Laboratory Animal Center, Zhejiang Academy of Medical Sciences.

The nude mice were then injected subcutaneously with LM3 cell suspensions. The mice were randomly grouped when the tumor reached a size of around 100 mm^3^. The mice were injected with G−LPQDEA/shSLC7A11 at an shSLC7A11 dose of 1.5 mg kg^−1^ in the treatment group. The tumor volumes were calculated as follows: V (mm^3^) = 0.5 × a × b^2^ (a: length; b: width). After 18 days of treatment, the nude mice were euthanized, and the tumor weights were recorded and tumor inhibition rates were calculated. Tumor samples, serum, and main organs (hearts, livers, spleens, lungs, kidneys) were harvested for a further analysis.

#### 2.2.12. Biodistribution Study

Fluorescent probe DID was encapsulated into G−LPQDEA/shSLC7A11 (G−LPQDEA/shSLC7A11/DiD) to track the biodistribution. LM3 cells were injected subcutaneously into the subcutaneous tissue on the right flank of nude mice. When the tumor volume reached ≈ 100 mm^3^, the mice were intravenously administered with G−LPQDEA/shSLC7A11/DiD at a DiD dose of 2 µg per mouse. Mice were sacrificed at predetermined time intervals. Tumors and the primary organs (hearts, livers, spleens, lungs, kidneys) were extracted and washed with saline and subjected to imaging using a near-infrared fluorescence (NIRF) imaging system (PerkinElmer IVIS Lumina XRMS Series III imaging system).

#### 2.2.13. Western Blotting

Cells or tissue lysates were added to a lysis buffer supplemented with protease and phosphatase inhibitors on ice for 30 min. Then, cell lysates were centrifuged at a speed of 12,000 rpm for 15 min at 4 °C and quantified using the Bicinchoninic Acid (BCA) method (Beyotime, Shanghai, China). After that, samples were diluted in a loading buffer separated on SDS-PAGE gels and then electro-blotted to polyvinylidene difluoride (PVDF, Roche, IN, USA) membranes and blocked with 5% milk in 0.1% Tween-TBS for 1.5 h at room temperature and then incubated with the primary antibodies overnight at 4 °C. The following antibodies were used: SLC7A11 (1:1000, Proteintech, Wuhan, China), GPX4 (1:1000, Proteintech, Wuhan, China), GAPDH (1:1000, Proteintech, Wuhan, China). PVDF membranes were washed three times using TBST and then immersed in the secondary antibody (1:10,000, Wuhan Boster, China) and incubated for 2 h at room temperature. The results were detected with an ECL system (Amersham Biosciences, Slough, UK) using an image reader. GAPDH was served as an internal control.

#### 2.2.14. RT-qPCR

Total RNA was extracted using a MolPure^®^ Cell/Tissue Total RNA Kit (YESEN, Shanghai, China), and cDNA was synthesized with Hifair^®^ III 1st Strand cDNA Synthesis SuperMix (YESEN, Shanghai, China). Quantitative real-time PCR was performed using SYBR GreenER qPCR SuperMix Universal according to the manufacturer’s protocol. The following primers were used:

GPX4 reverse—5′-GCAGCCGTTCTTGTCGATGAGG-3′

GPX4 forward—5′-CCGCTGTGGAAGTGGATGAAGATC-3′

SLC7A11 forward—5′-GGCTCCATGAACGGTGGTGTG-3′

SLC7A11 reverse—5′-GCTGGTAGAGGAGTGTGCTTGC-3′

β-Actin forward—5′-CATGTACGTTGCTATCCAGGC-3′

β-Actin reverse—5′-CTCCTTAATGTCACGCACGAT-3′

#### 2.2.15. In Vivo Safety Evaluation

After four intravenous injections, the whole blood and serum were harvested and subjected to an analysis.

#### 2.2.16. Statistical Analysis

An unpaired Student’s *t*-test was utilized to conduct statistical comparisons between the two groups. To identify significant differences among multiple groups, a one-way analysis of variance (ANOVA) was used, followed by a *t*-test and Kruskal–Wallis test. A survival analysis was performed based on a Kaplan–Meier analysis. The data are displayed as means ± standard deviation (S.D.). Prism 8.0 software (GraphPad Software, San Diego, CA, USA) was utilized for conducting all statistical analyses.

## 3. Results

### 3.1. SLC7A11 Expression Was Associated with Progression and Prognosis of Human HCC

**Figure 1:**
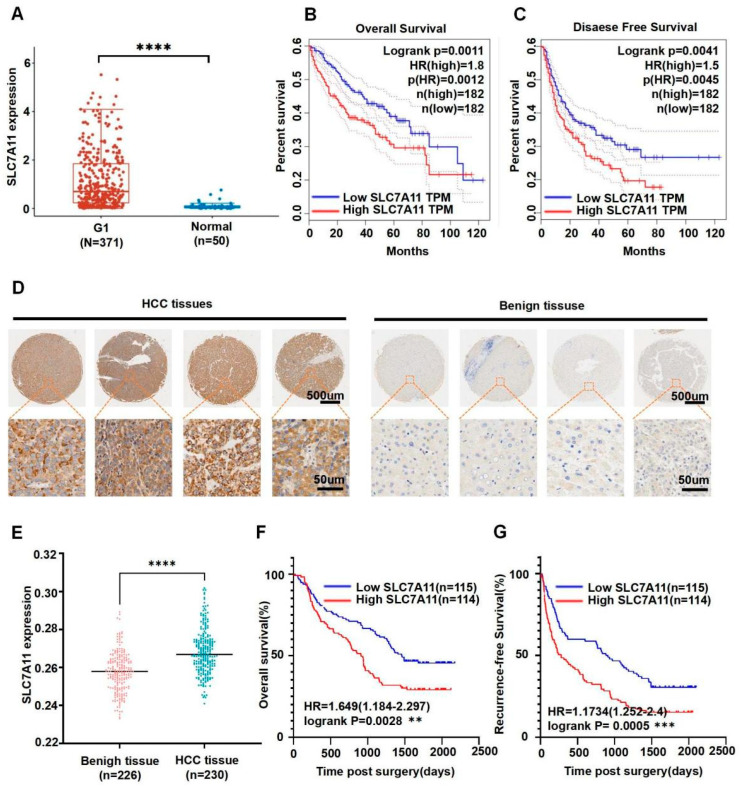
Correlation analysis of HCC prognosis with SLC7A11 expression. (**A**) SLC7A11 expression in HCC tissues and normal liver tissues. Data were collected from TCGA database. Progression-free survival (PFS) (**B**) and overall survival (OS) (**C**) were analyzed with Kaplan–Meier method. (**D**) Representative immunohistochemical images of tissue microarrays stained with SLC7A11. Scale bars: 500 μm and 50 μm. (**E**) Quantitative analysis of SLC7A11 expression with immunohistochemistry (IHC) staining analysis. (**F**,**G**) The overall survival (OS) and recurrence-free survival (RFS) of patients categorized by low/high SLC7A11 expression based on IHC analysis. The data are represented as mean ± SD; ** *p* < 0.01, *** *p* < 0.001, **** *p* < 0.0001.

The TCGA database showed that HCC tissues exhibited notably elevated levels of SLC7A11 compared to benign liver tissues (Figure 1A). In addition, we noticed a clear correlation between elevated SLC7A11 levels with recurrence-free survival (RFS) and overall survival (OS) (Figure 1B,C).

Subsequently, adjacent normal liver tissues (*n* = 226) and HCC tissues (*n* = 230) were incorporated into tissue microarrays (TMAs) and subjected to an SLC7A11 expression analysis. HCC tissues exhibited significantly higher expression levels of SLC7A11 compared with benign liver tissues (Figure 1D,E). We observed a consistent tendency, which indicated that elevated SLC7A11 expression levels were associated with overall survival and recurrence-free survival, and the differences were statistically significant (Figure 1F,G), suggesting that SLC7A11 is a potential prognostic biomarker that contributes to the progression of HCC.

### 3.2. Construction of Polyplexes

**Figure 2:**
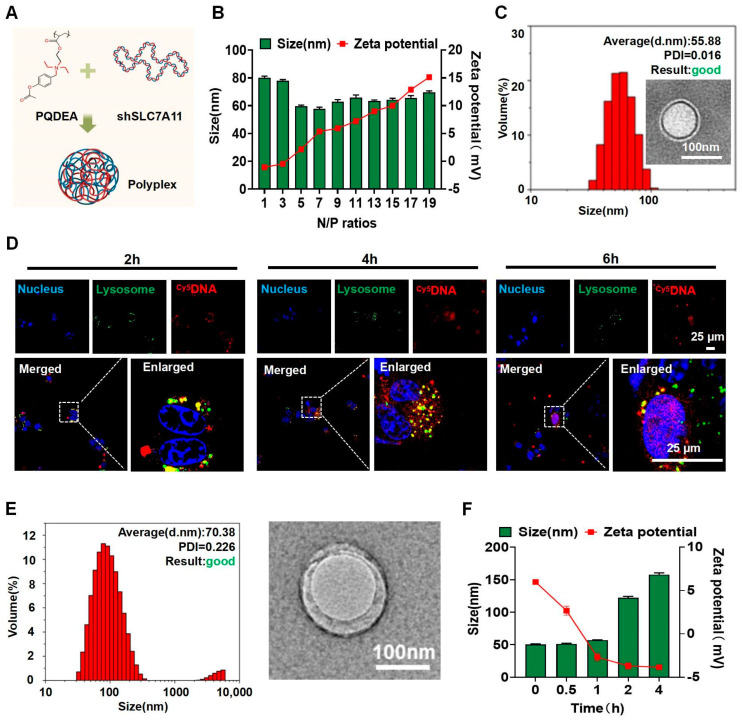
Characterization of cationic liposomal-coated G−LPQDEA/shSLC7A11 nanoparticles. (**A**) Illustration of PQDEA/shSLC7A11 fabrication. (**B**) The sizes and corresponding zeta potentials of the PQDEA/shSLC7A11 polyplexes with various N/P ratios. (**C**) The optimized size distribution of the polyplexes (N/P = 7) measured through dynamic laser scattering (inserted: TEM image). (**D**) Intracellular trafficking of PQDEA/shSLC7A11 polyplexes in LM3 cells observed via confocal microscopy at different times. Hoechst 33342 was used to stain the cell nuclei, appearing in a blue color, and Cy^5^ was used to label the shSLC7A11, which is shown in red. Lysotracker Green was used to stain the lysosomes, which are depicted. (**E**) Size distribution and TEM image of G−LPQDEA/shSLC7A11. (**F**) Stabilities of G−LPQDEA/shSLC7A11 nanoparticles in terms of sizes and zeta potentials at different times after incubation in medium containing 10% serum.

The PQDEA was synthesized through the quaternarization of poly[2-(N,N-diethylamino)ethyl acrylate] (PDEA) with 4-(chloromethyl)phenyl acetate. This cationic PQDEA effectively complexed anionic SLC7A11 shRNA (shSLC7A11) through the electrostatic interactions, yielding uniform particles with a consistent size of around 65 nm, offering distinct advantages for endocytosis and tumor penetration. The PQDEA polyplexes’ zeta potentials ranged from −1 mV to +15 mV (Figure 2A–C).

The PQDEA polyplexes’ transfection mechanism was elucidated with confocal microscopy. The rapid cellular uptake of Cy^5^-shSLC7A11 polyplexes was evident; after 2 h of incubation, the shSLC7A11 entered the LM3 cells and migrated toward the nucleus. Notably, a fraction of the shSLC7A11 appeared to be associated with lysosomes (red and green overlap forming yellow dots). However, a significant portion of shSLC7A11 (red) had already disengaged from lysosomes (green), proceeding to enter the nucleus. At 4 h, a substantial amount of shSLC7A11 had entered the nucleus. At 6 h, the nanocomplexes transitioned from their initial dot-like configuration (indicative of partial PQDEA and shSLC7A11 dissociation) to a more diffused form. Most shSLC7A11 was now free and fully disassociated from the polymer. In summary, the PQDEA/shSLC7A11 polyplexes exhibited rapid cellular internalization and subsequent nuclear entry (Figure 2D).

To prevent esterase-caused dissociation before entering cancer cells, the PQDEA/shSLC7A11 polyplexes were then coated with a pegylated lipid layer. The optimized molar ratio of cationic lipid DOTAP/DOPC to PQDEA monomer units was determined to be 1:3:3 for PQDEA/shSLC7A11 polyplexes with an N/P ratio of 7.

To meet with the demands of intravenous administration and targeted delivery to liver cells, 10% of galactose-DSPE-PEG and DSPE-PEG (molar ratio, 1:1) were incorporated to shield the cationic charges and stabilize the lipid-coated polyplexes, ultimately termed “G−LPQDEA/shSLC7A11”, with a size of around 70 nm (Figure 2E). The G−LPQDEA/shSLC7A11 demonstrated robust stability in the solution, even when serum was present. PQDEA/shSLC7A11 and G−LPQDEA/shSLC7A11 were incubated simultaneously in the serum-containing medium. After incubation, the zeta potential of PQDEA/shSLC7A11 changed from positive to negative after 1 h of incubation, and the particle size also increased gradually over time, reaching 300 nm after incubation for 2 h. After a 4 h incubation, the PQDEA/shSLC7A11 composite had essentially vanished, and the nanocomplex gradually disassembled (Figure S2). In contrast, although the surface potential of G−LPQDEA/shSLC7A11 also changed from positive to negative after incubation in the serum-containing medium, the particle size of the complex remained stable at about 150 nm even at 4 h post incubation, indicating that G−LPQDEA/shSLC7A11 was stable in serum (Figure 2F).

### 3.3. G−LPQDEA/shSLC7A11 Reduced the Viability of HCC Cell Lines

**Figure 3:**
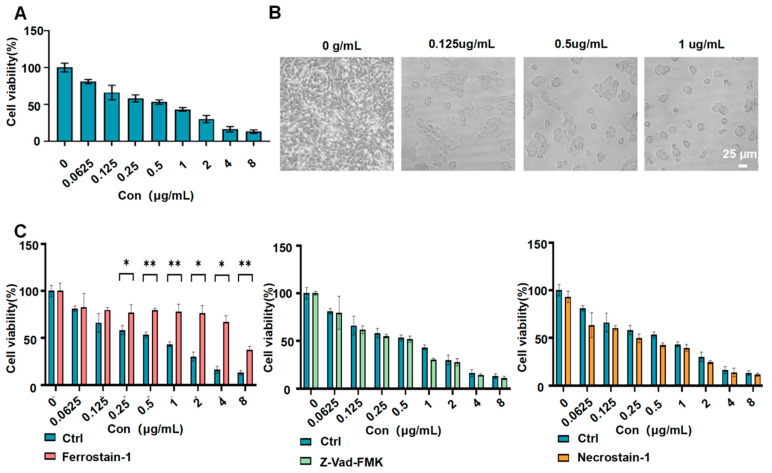
G−LPQDEA/shSLC7A11 reduced the viability of LM3 cells. (**A**) Different concentrations of G−LPQDEA/shSLC7A11 (ranging from 0.025 to 8 μg/mL) were administered to LM3 cells for a duration of 48 h. After 48 h, the vitality of the cells was assessed. The results indicated that G−LPQDEA/shSLC7A11 decreased LM3 viability in vitro in a dose-dependent manner. Data are represented as mean ± standard deviation. (**B**) Cell morphology after being exposed to various concentrations of G−LPQDEA/shSLC7A11 (ranging from 0.025 to 8 μg/mL) for 48 h. (**C**) LM3 was treated with different concentrations of G−LPQDEA/shSLC7A11 (0.025–8 μg/mL) with or without Necrostain-1, Z-Vad-FMK, and Ferrostatin-1 for 48 h. The cell viabilities were assessed with CCK-8 assay. The results also revealed that neither the necrosis inhibitor Necrostain-1 nor the apoptosis inhibitor Z-Vad-FMK could rescue cell viabilities that had been inhibited by G−LPQDEA/shSLC7A11. The findings indicated that the cell viability, which was suppressed by G−LPQDEA/shSLC7A11, was rescued by the ferroptosis inhibitor Ferrostatin-1. Data are represented as mean ± SD; * *p* < 0.05, ** *p* < 0.01.

We also examined cytoxicities of G−LPQDEA/shSLC7A11 LM3 cells using the CCK-8 assay (Figure 3A). Treatment with varying concentrations of shSLC7A11 revealed a dose-dependent inhibition in LM3 cell viability. The calculated IC_50_ value for G−LPQDEA/shSLC7A11 in LM3 cells was 2.15 μg/mL (in terms of shSLC7A11 dose). Meanwhile, the CCK8 assay showed a decrease in cytotoxicity on the normal liver cell line AML12 (IC _50_ = 4.58 μg/mL) relative to LM3 cells (Figure S3). Notably, treatment of LM3 cells with the same proportion of carriers without sh SLC7A11 did not exhibit any significant decrease in cell viability (Figure S4). The microscopic examination of LM3 cells treated with G−LPQDEA/shSLC7A11 revealed distinct changes in cell morphology, including fragmentation, transformation, and multi-directional alterations (Figure 3B).

To explore whether the viability inhibition with G−LPQDEA/shSLC7A11 was associated with ferroptosis, LM3 cells were exposed to different doses of shSLC7A11 (ranging from 0.025 to 8 μg/mL) with either Ferrostatin-1, a ferroptosis inhibitor, Z-Vad-FMK, an apoptosis inhibitor, or Necrostatin-1, a necrosis inhibitor. Cell viabilities were successfully rescued by Ferrostatin-1 in the presence of G-LPQDEA/shSLC7A11, whereas neither Z-Vad-FMK nor Necrostatin-1 were able to carry out this (Figure 3C).

### 3.4. G−LPQDEA/shSLC7A11 Induced ROS-Mediated Ferroptosis of HCC Cell Lines

**Figure 4:**
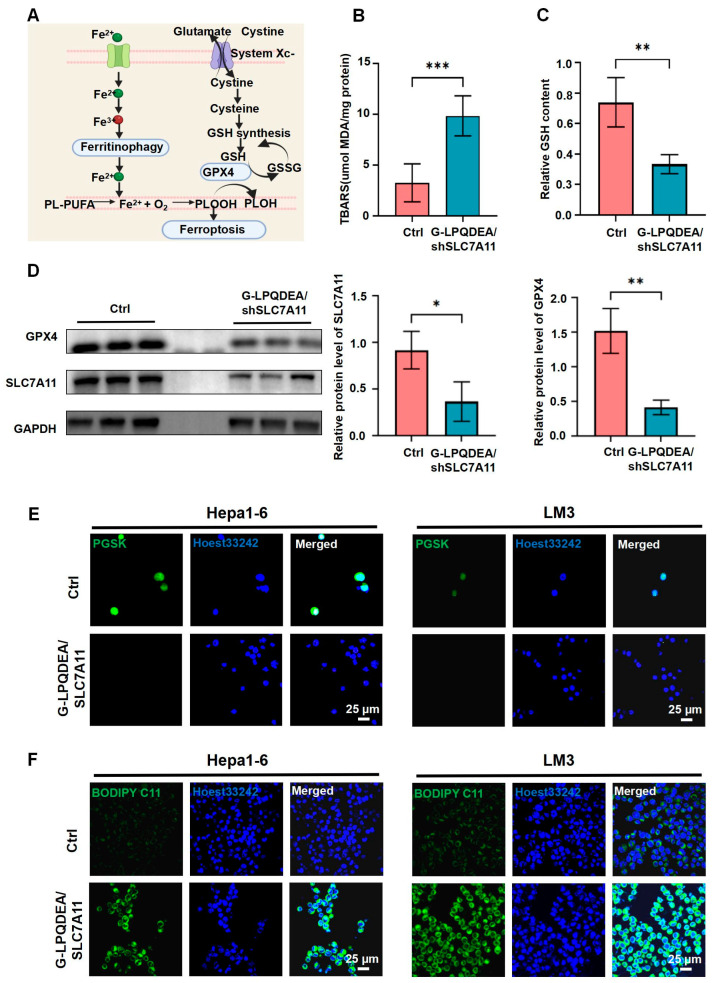
G−LPQDEA/shSLC7A11 transfection induced ROS-mediated ferroptosis of HCC cell lines. (**A**) Illustration of ferroptosis mechanisms. When system Xc− is inhibited, it causes a decrease in GSH levels and then inactivated GPX4. This ultimately leads to the buildup of harmful lipid peroxides and triggers ferroptosis. Excessive amounts of Fe^2+^ cause lipid ROS accumulation through the Fenton reaction, leading to ferroptosis. (**B**) The comparative analysis of relative MDA contents was conducted between the control and G−LPQDEA/shSLC7A11 treatment groups. (**C**) The comparison of GSH contents between the control group and the G−LPQDEA/shSLC7A11 group. (**D**) Western blot analysis of SLC7A11 protein and GPX4 protein expression. The grayscale of each band was measured using Image J 1.47v program and standardized to the corresponding GADPH intensity. (**E**) Fluorescence imaging of PGSK in LM3 and Hepa 1–6 cells after being treated with G−LPQDEA/shSLC7A11 for 48 h. (**F**) Confocal imaging analysis of ROS levels in LM3 or Hepa 1–6 cells treated with or without G−LPQDEA/shSLC7A11. Data are represented as mean ± SD; * *p*  <  0.05, ** *p* < 0.01, *** *p*  < 0.001.

We then examined the characteristic manifestations of the ferroptosis process (Figure 4A). Lipid peroxide accumulation (malondialdehyde, MDA) is one of the main features of ferroptosis [31,32]. As shown in Figure 4B,C, the MDA levels were higher in G−LPQDEA/shSLC7A11-treated cells compared to the control group, accompanied by reduced GSH levels, indicating that G−LPQDEA/shSLC7A11 can induce ferroptosis in HCC cells via lipid peroxidation.

SLC7A11 and GPX4 are two key proteins in the ferroptosis pathway [33]. Therefore, the expression of these two proteins was analyzed. As seen from Figure 4D, G−LPQDEA/shSLC7A11-treated LM3 cells had a relatively low expression of SLC7A11 and GPX4 compared to the control groups.

We further evaluated Fe^2+^ concentration and found a significant increase (PGSK intensity decreased) after 48 h of incubation with G−LPQDEA/shSLC7A11 (Figure 4E). Remarkably, shSLC7A11-treated LM3 cells significantly induced intracellular lipid ROS accumulation (Figure 4F). Collectively, the above investigations suggest that G−LPQDEA/shSLC7A11 can induce ferroptosis of HCC cells.

### 3.5. G−LPQDEA/shSLC7A11 Could Inhibit HCC Growth and Prolong Mouse Survival with Minimal Toxicity

**Figure 5:**
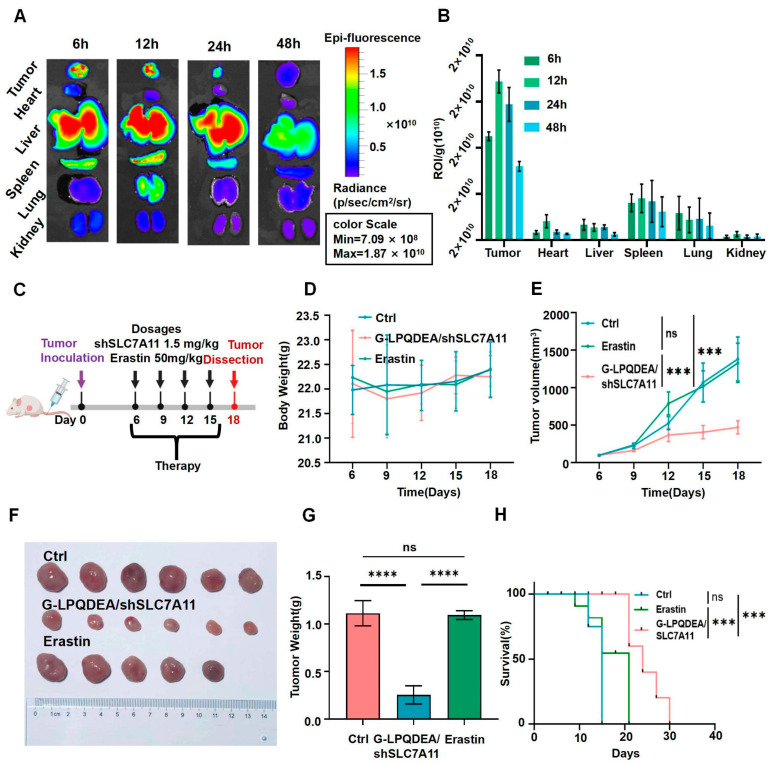
G-LPQDEA/SLC7A11 inhibited LM3 tumor growth in vivo. (**A**,**B**) Mice were injected with DID-loaded G-LPQDEA/shSLC7A11 (DiD, 0.75 mg kg^−1^). At 12, 24, 48, or 72 post injection, tumors and major organs (*n* = 3) were dissected and imaged. The intensities were quantitated. (**C**) On Day 6, the G−LPQDEA/shSLC7A11 group was initiated using i.v. injection on an every-three-day schedule (shSLC7A11 dose, 1.5 mg kg^−1^), and the erastin group was intraperitoneally injected at a dose of 50 mg kg^−1^. (**D**) Body weight curves of mice in the experimental period. (**E**) Tumor growth curves. (**F**) The dissected tumor images at the endpoint. (**G**) Tumor weight was determined and compared between the G−LPQDEA/shSLC7A11 treatment with erastin and control groups. (**H**) Survival curves of mice receiving different treatments in (**D**). Data are represented as mean ± SD; *** *p* < 0.001,**** *p* < 0.0001, ns, not significant.

A biodistribution study was conducted on xenograft nude mice bearing LM3 tumors. DiD was loaded into G−LPQDEA/shSLC7A11 and imaged at various time points post-injection. At 12, 24, 48, and 72 h post injection, tumors and main organs were dissected for imaging (Figure 5A). DiD-loaded G−LPQDEA/shSLC7A11 effectively accumulated in tumors, displaying robust signals even up to 72 h post injection (Figure 5B). At each time point, the fluorescence intensity in tumors was notably higher than that in organs.

To evaluate in vivo therapeutic efficacy, tumor volumes of G−LPQDEA/shSLC7A11 and erastin-treated mice were traced using the regime described in Figure 5C. Erastin is the ferroptosis inducer most commonly used in research studies [34]. G−LPQDEA/shSLC7A11 was i.v. injected at an shSLC7A11 dose of 1.5 mg kg^−1^, and 50 mg kg^−1^ of erastin was intraperitoneally injected according to a treatment regimen initiated on Day 6 post-inoculation. The mice were sacrificed on Day 3 post the last treatment, and the tumors were collected and analyzed (Figure 5D).

As shown in Figure 5E,F, tumor volumes and weights were substantially reduced in the G−LPQDEA/shSLC7A11 treatment group, with an average inhibition rate of 77% compared to the saline control (*p* < 0.0001), which is also significantly lower than erastin-treated mice. Mouse body weight curves showed that G−LPQDEA/shSLC7A11 treatment did not exhibit weight loss (Figure 5G).

The prognosis of the G−LPQDEA/shSLC7A11 therapy is further evaluated with the survival study (Figure 5H). The overall survival of mice was 15 days in the control group and extended to 21 days for erastin-treated mice. G−LPQDEA/shSLC7A11 treatments significantly prolonged mouse survival to 30 days, as shown in Figure 5H.

**Figure 6:**
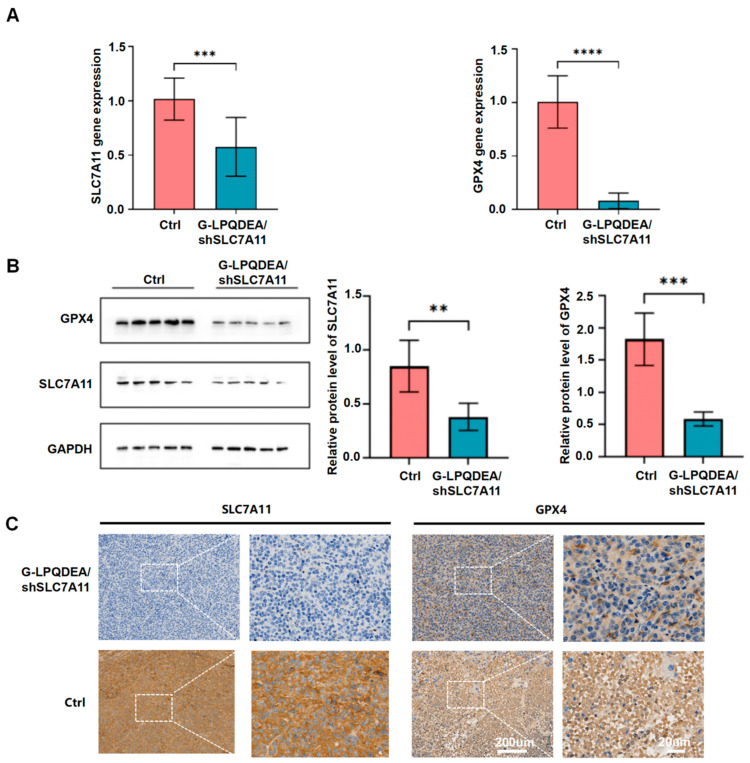
G−LPQDEA/shSLC7A11 treatments downregulated SLC7A11 and GPX4 expression in tumors. (**A**) Relative mRNA expression levels of SLC7A11 and GPX4 in G−LPQDEA/shSLC7A11-treated tumors and control group tumors. (**B**) Western blot analysis of SLC7A11 and GPX4 protein expression. Image J 1.47v software was used to quantify each band’s grayscale and normalize it to the corresponding GAPDH intensity. (**C**) Immunohistochemical analysis of SLC7A11 and GPX4 expression in tumor samples. The tissue paraffin sections were 5 μm thick. Data are represented as mean ± SD; ** *p* < 0.01, *** *p* < 0.001,**** *p* < 0.0001.

The tumor sample analysis showed that SLC7A11 and GPX4 mRNA expression were notably decreased in G−LPQDEA/shSLC7A11-treated tumors compared to the control group (Figure 6A). Western blotting and the IHC analysis also confirmed a remarkable reduction in SLC7A11 and GPX4 protein expression in the G−LPQDEA/shSLC7A11-treated group compared to the control (Figure 6B,C).

**Figure 7:**
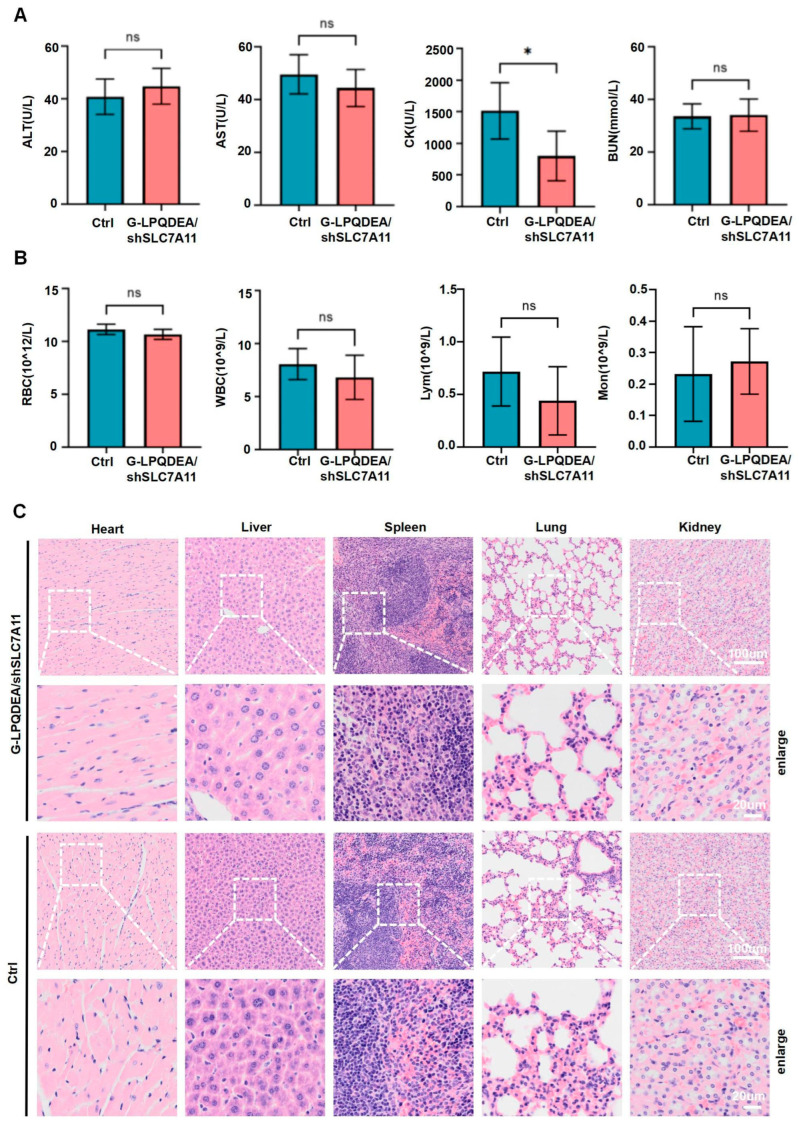
Biosafety evaluation. (**A**) Serum biochemistry analysis after G−LPQDEA/shSLC7A11 treatments (*n* = 5). (**B**) Blood routine levels after G−LPQDEA/shSLC7A11 treatments (*n* = 5). (**C**) Histomorphology of the liver, spleen, kidney, lung, spleen, and heart analyzed via hematoxylin and eosin (HE) staining. Data are represented as mean ± SD; * *p*  <  0.05. ns, not significant.

Hepatorenal indexes, e.g., alanine aminotransferase (ALT), aspartate aminotransferase (AST), blood urea nitrogen (BUN), creatine kinase (CK), and blood routine, e.g., red blood cells (RBCs), white blood cells (WBCs), lymphocytes (Lym), and monocytes (Mon), were also assessed, together with HE staining analyses of major organs (liver, spleen, lung, kidney, and heart) (Figure 7A,B). Compared with the control group, G−LPQDEA/shSLC7A11 treatments exhibited no obvious abnormal changes (Figure 7C). All together, these results demonstrated that G-LPQDEA/shSLC7A11 strongly suppresses tumor growth in vivo with negligible toxicities.

## 4. Discussion

HCC is ranked the sixth most aggressive tumor worldwide [35], with a 5-year survival rate of less than 20% [36]. Conventional treatments have limited therapeutic outcomes and there is an urgent need for developing effective therapies with minimal side effects.

Generally speaking, chemotherapeutic drugs usually cause cancer cell death by inducing apoptosis [9,10]. Ferroptosis, characterized by mitochondrial damage, is a novel type of cell death identified in 2012 and has shown promise as a therapeutic option for the treatment of HCC [12,13,14,15]. Studies have revealed that many small-molecule therapeutic agents, such as sorafenib, erastin, sulfasalazine, et al., are effective in inducing cancer cell ferroptosis [17,18,19,20,21]. Currently, the main mechanism of ferroptosis is the SLC7A11-GSH-GPX4 axis.

SLC7A11 is an essential part of system Xc, which is responsible for orchestrating the influx of cystine while exporting glutamate, leading to the production of GSH, which interacts with GPX4 to decrease PL-OOH and produce the corresponding P-LOH. Ultimately, this helps to protect the integrity of cell membranes and inhibit ferroptosis [12]. Recent studies have revealed that SLC7A11 is overexpressed in various cancer types, and promotes cancer progression by inhibiting ferroptosis [37], e.g., lung cancer [38], breast cancer [39], bladder cancer [40], et al., demonstrating it as a potential therapeutic target. In this study, we also observed that the expression level of SLC7A11 in tumor tissue of patients with HCC was significantly higher than adjacent benign tissue, as evident with the TCGA database and tissue samples from patients with HCC. Furthermore, high SLC7A11 expression levels in HCC tissues were correlated with an unfavorable prognosis. It has been reported that the downregulation of SLC7A11 could decrease intracellular cystine and GSH levels, which in turn suppresses GPX4 activity and induces ferroptosis [19,23], as also demonstrated in HCC cells. However, these small molecules are easily degraded and have low tumor-targeting efficiency, limiting the therapeutic outcome.

To address the above issues, we developed an esterase-responsive cationic polymer-based gene delivery system (G−LPQDEA/shSLC7A11) for downregulating SLC7A11 expression. PQDEA’s susceptibility to intracellular esterase hydrolyzation facilitated its transformation into a negatively charged poly (acrylic acid), resulting in shSLC7A11 release and subsequently inhibited SLC7A11 transcription and expression [28,29,30]. Meanwhile, the introduction of a galactose-decorated lipid layer increased the blood circulation time of shSLC7A11 and guaranteed HCC-specific delivery of shSLC7A11. In the present study, we evaluated the anti-cancer efficacy of G−LPQDEA/shSLC7A11 in an HCC subcutaneous tumor model. G−LPQDEA/shSLC7A11 effectively retarded tumor development and prolonged survival compared to the control and conventional ferroptosis inducer, erastin, with negligible toxicities.

In summary, the G−LPQDEA/shSLC7A11 delivery system exhibited potent antitumor efficiency via ferroptosis induction and is expected to be promising for HCC treatment.

## Data Availability

The data can be shared up on request.

## Supplementary Materials

The following supporting information can be downloaded at: https://www.mdpi.com/article/10.3390/pharmaceutics16020249/s1. Figure S1. The plasmid map that codes for shSLC7A11 plasmid; Figure S2. Stabilities of PQDEA/shSLC7A11 NPs; Figure S3. AML12 cells were treated with different concentrations of G−LPQDEA/shSLC7A11 (0.025–8 μg/mL). The cell viability was determined by the CCK8 assay (n = 3; incubation time: 48 h); Figure S4. LM3 cell viabilities determined by CCK8 assay after incubation with blank NPs (n = 3; incubation time: 48 h). Figure S5. Live imaging of DiD-loaded G−LPQDEA/shSLC7A11 in the LM3 tumors and main organs of the mice after i.v. injection (DiD, 0.75 mg kg^−1^); Supplementary Table S1. Whole blood cell analysis; Supplementary Table S2. Serum biochemistry analysis; Supplementary Table S3. Antibodies used in this study; Supplementary Table S4. Primers used in this study.
