# Supplementary material for: An Esterase-Responsive SLC7A11 shRNA Delivery System Induced Ferroptosis and Suppressed Hepatocellular Carcinoma Progression
Source: Pharmaceutics. 2024 Feb 8;16(2):249. doi: 10.3390/pharmaceutics16020249 (PMC10891814; doi:10.3390/pharmaceutics16020249)
Supplement: Supplementary file 1 [file pharmaceutics-16-00249-s001.zip › pharmaceutics-2797994-supplementary.pptx]

## Slide 1
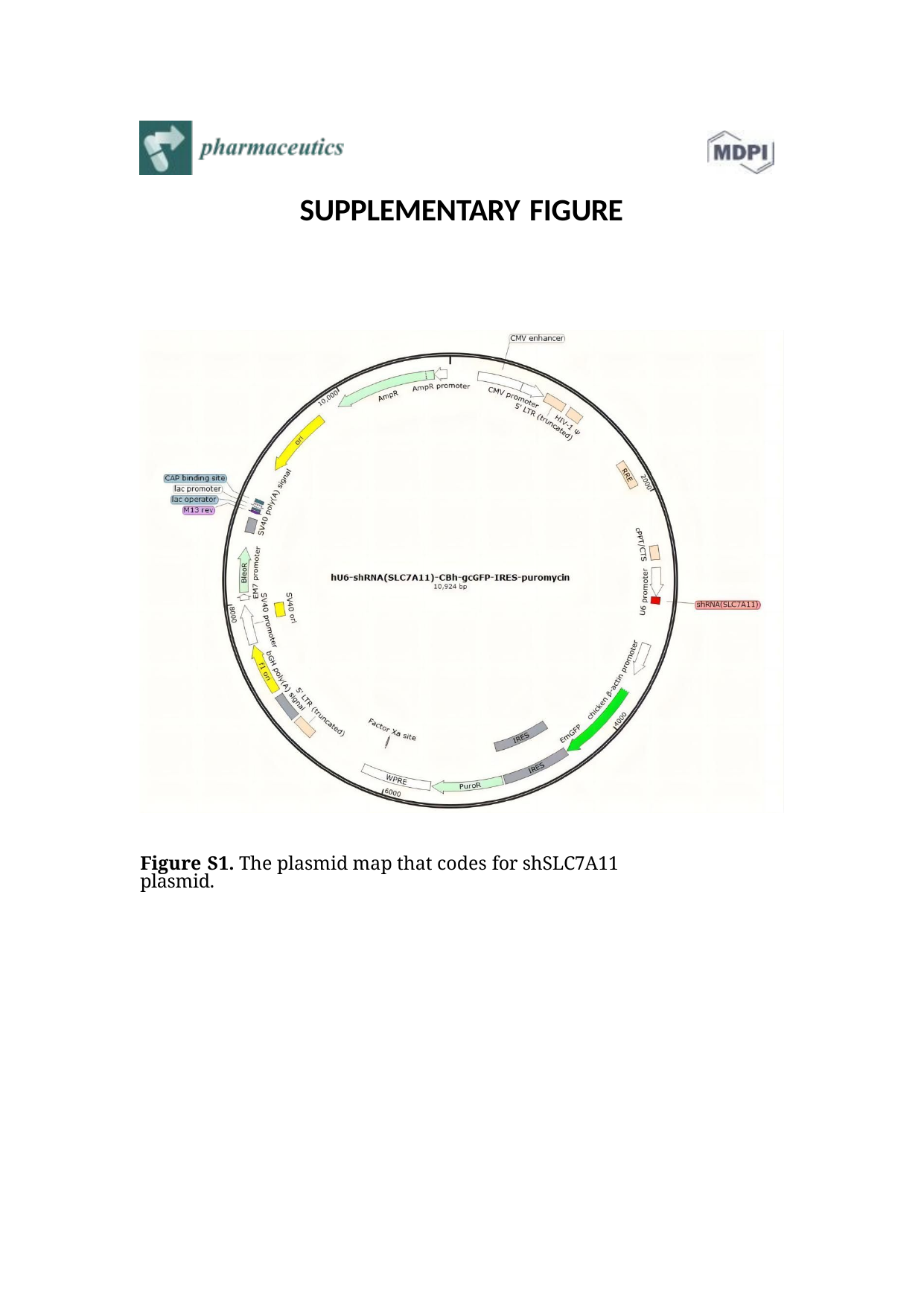

SUPPLEMENTARY FIGURE
Figure S1. The plasmid map that codes for shSLC7A11 plasmid.

## Slide 2
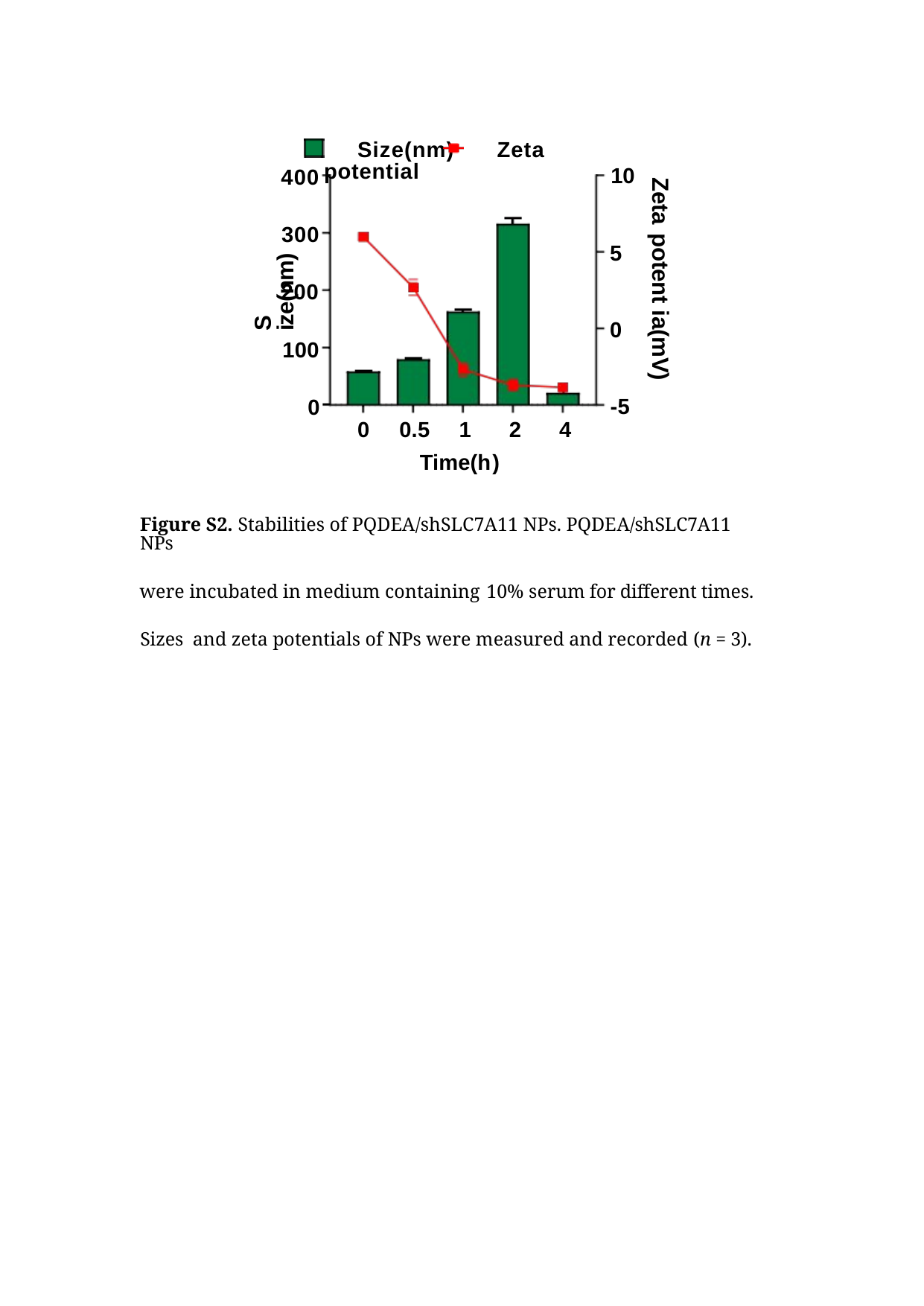

Size(nm) Zeta potential
10
5
0
-5
400
300
200
100
0
Zeta potent ia(mV)
S ize(nm)
0 0.5 1 2 4
Time(h)
Figure S2. Stabilities of PQDEA/shSLC7A11 NPs. PQDEA/shSLC7A11 NPs
were incubated in medium containing 10% serum for different times. Sizes and zeta potentials of NPs were measured and recorded (n = 3).

## Slide 3
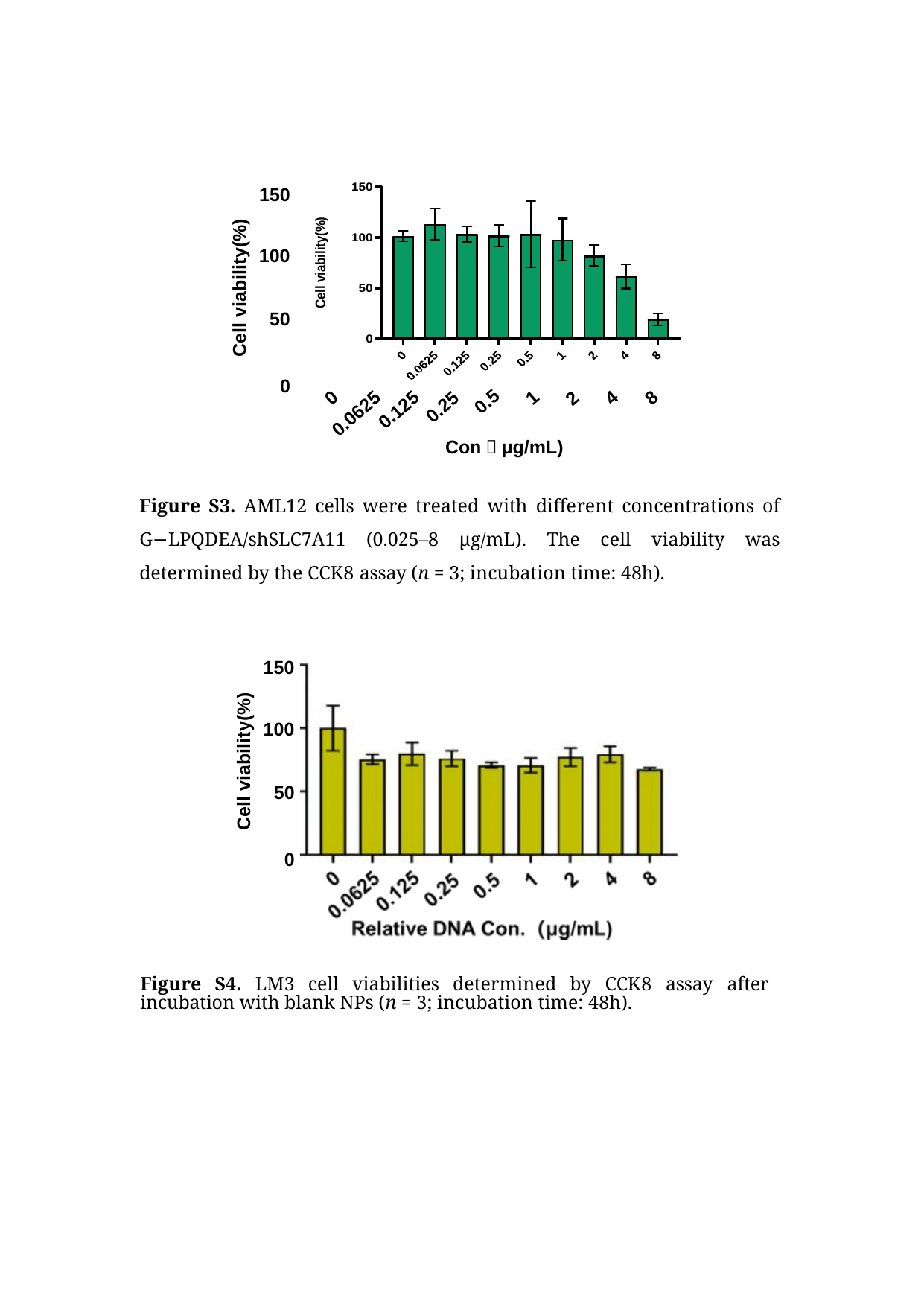

150
100
Cell viability(%)
 50
 0
0.5
4
0.0625
0.125
1
8
0.25
2
0
Con（μg/mL)
Figure S3. AML12 cells were treated with different concentrations of G−LPQDEA/shSLC7A11 (0.025–8 μg/mL). The cell viability was determined by the CCK8 assay (n = 3; incubation time: 48h).
150
100
Cell viability(%)
 50
 0
Figure S4. LM3 cell viabilities determined by CCK8 assay after incubation with blank NPs (n = 3; incubation time: 48h).

## Slide 4
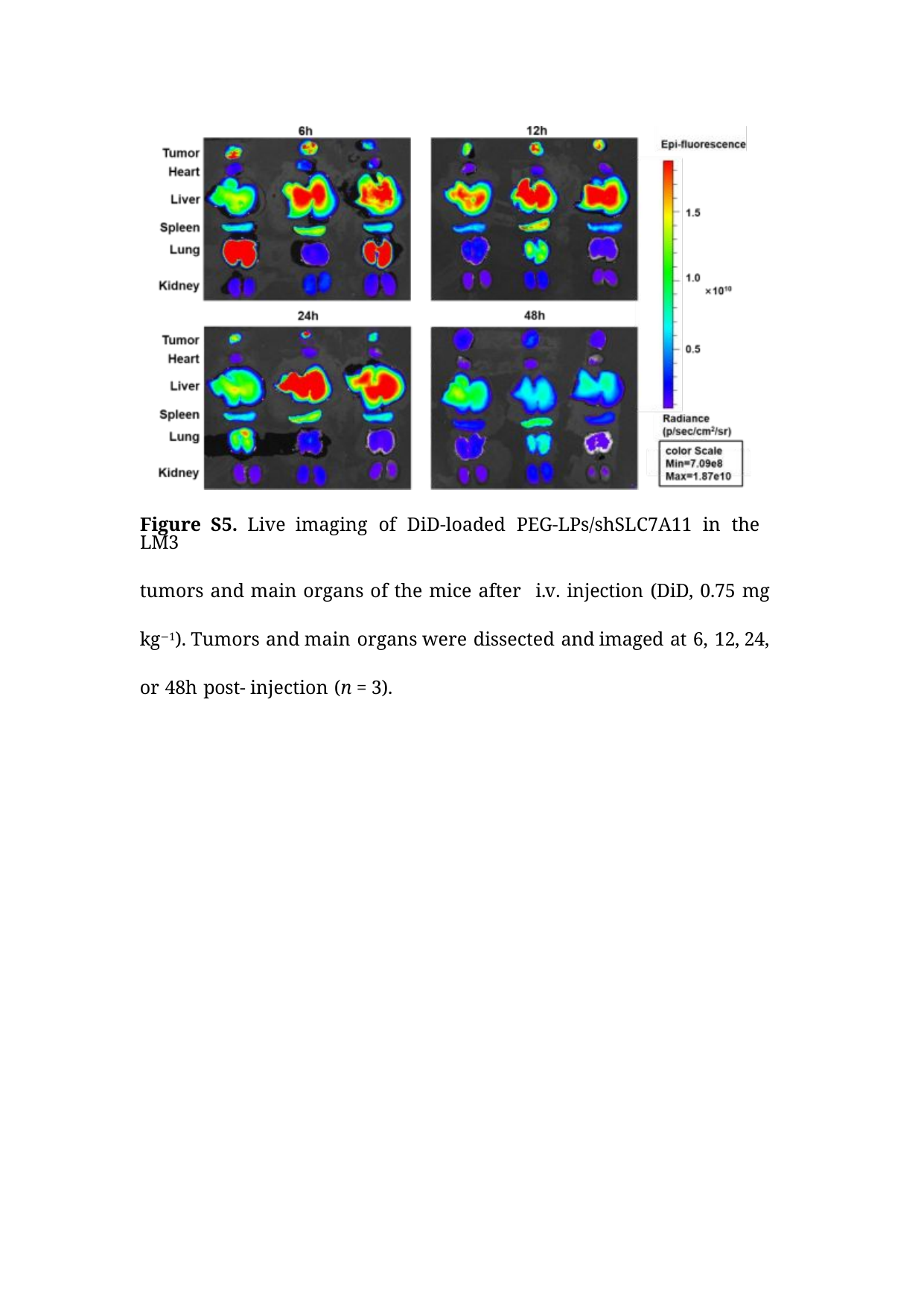

Figure S5. Live imaging of DiD-loaded PEG-LPs/shSLC7A11 in the LM3
tumors and main organs of the mice after i.v. injection (DiD, 0.75 mg kg−1). Tumors and main organs were dissected and imaged at 6, 12, 24, or 48h post- injection (n = 3).

## Slide 5
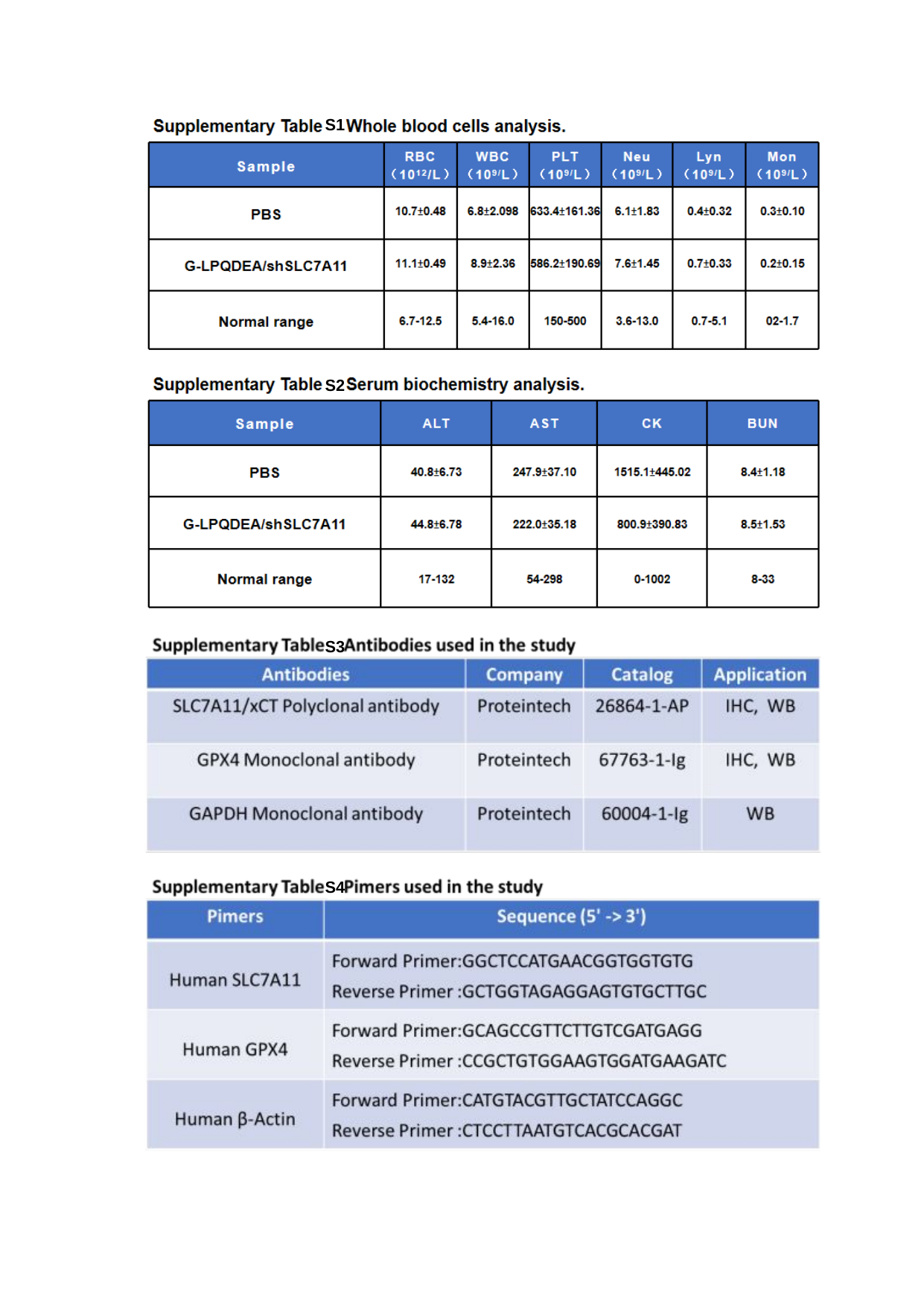

S1
S2
S3
S4
